# Supplementary figures and images for: ViralPlaque: a Fiji macro for automated assessment of viral plaque statistics
Source: PeerJ. 2019 Sep 24;7:e7729. doi: 10.7717/peerj.7729 (PMC6764358; doi:10.7717/peerj.7729)

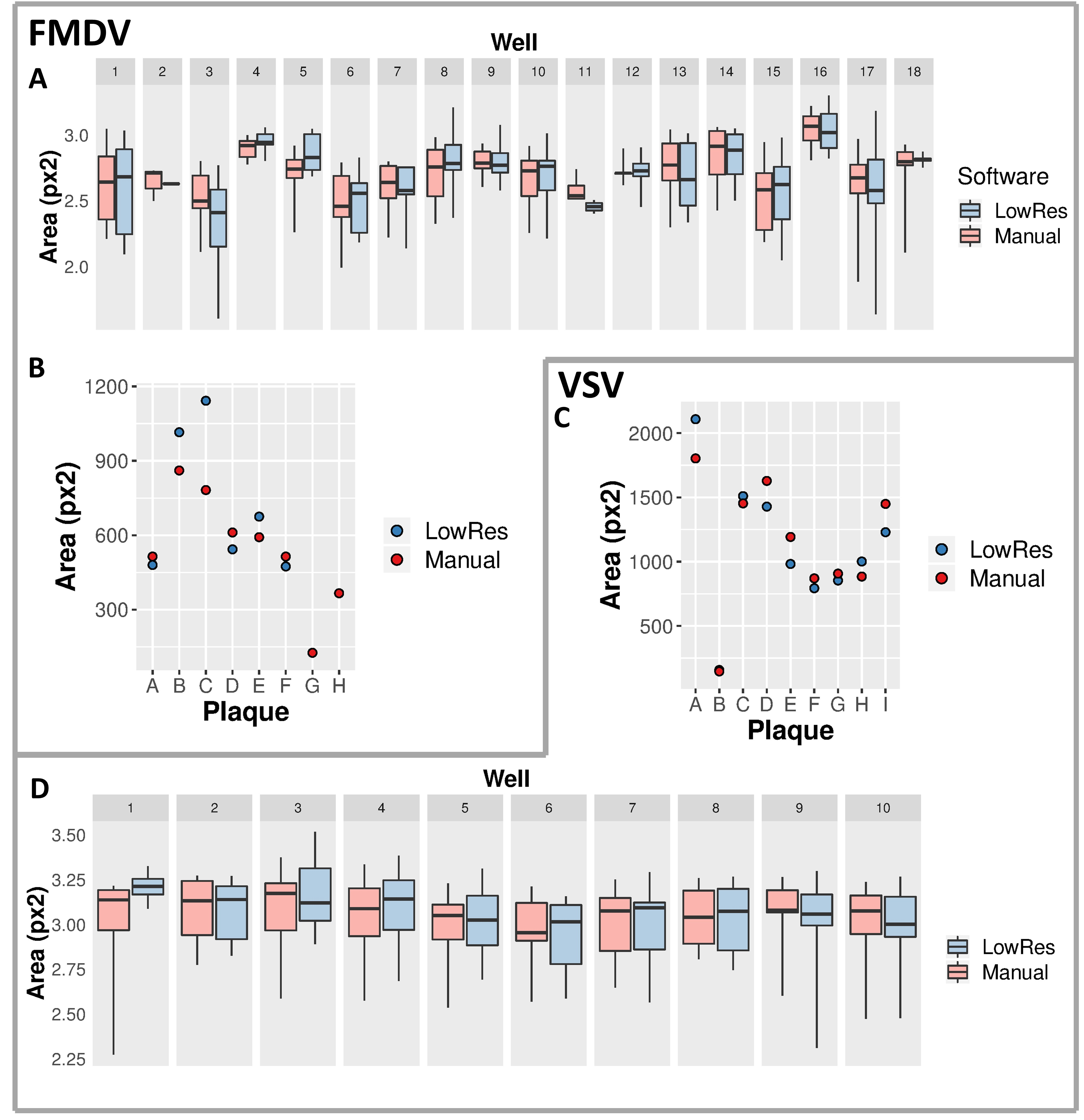

Supplement: Figure S1 — (A, D) Boxes represent distribution of data between 10th and 90th percentile; horizontal lines indicate median values. (B, C) Comparison of manual and automated measurement of plaque area for representative lysis plaques. In (B), plaque C (area = 1,142 px2 as detected by ViralPlaque) actually represents two lysis plaques (C and H) with areas of 782 and 366 px2 that were erroneously recorded as a single plaque. Plaques G and H were not detected by the IJ macro. [file peerj-07-7729-s001.png]
