# Supplementary material for: ViralPlaque: a Fiji macro for automated assessment of viral plaque statistics
Source: PeerJ. 2019 Sep 24;7:e7729. doi: 10.7717/peerj.7729 (PMC6764358; doi:10.7717/peerj.7729)
Supplement: File S1 [file peerj-07-7729-s002.docx]

Instructions for downloading and installing the macro ViralPlaque

1. Download and install Fiji (imageJ):

<https://fiji.sc/#download>

1. Download the macro ViralPlaque:

https://sourceforge.net/projects/viralplaque/

1. To install the macro: Plugins 🡪 Macros 🡪 Install

Navigate to and select the macro file, and click ‘Open’

Instructions for running the macro ViralPlaque (method LowRes)

1. Open image file to analyze.
2. To run the macro: Plugins 🡪 Macros 🡪 ViralPlaque
3. Enter settings values in popup (if none is changed default values are used):
   1. Enter number of pixels per unit. If scale is not known, leave as 0 and unit as pixel
   2. Define unit (px or mm)
   3. Enter min and max plaque size and minimum circularity.
   4. Enhance value can be changed (affects only Difference method).
   5. If Auto-thresholding box is checked step 6 will be omitted (affects only LowRes and Difference methods).
   6. If manual control box is unchecked step 7 will be omitted.
   7. If Count only box is checked only number of plaques detected will be informed. This option is designed specifically to count plaques so some parameters will be overwritten to specific values: Auto-thresholding=true; manual control=false; min plaque size = 40; minimum circularity = 0.5. This option is only available for LowRes method.
4. Use oval selection tool to select the well area to analyze.
5. Enter radius values for Median and Gaussian Blur filters in popup (if none is changed default values are used). Then press OK.

Note: Median radius should not be larger than the diameter of the smallest plaque to detect. Gaussian Blur radius should be approximately a quarter of the Median radius.

1. Use threshold window to select the optimal segmentation and press apply. Then press OK.
2. Use ROI manager to control the plaques detected:
3. to discard false positives, select them and press delete.
4. to manually add more plaques, select them using the oval selection tools and press add.
5. when finished, press measure in the ROI manager window.
6. View and save results.

Instructions for running the macro ViralPlaque (method Difference)

1. Open image file to analyze.
2. To run the macro: Plugins 🡪 Macros 🡪 ViralPlaque
3. Select method Difference in popup. Additionally, enter settings values in popup (if none is changed default values are used):
   1. Enter number of pixels per unit. If scale is not known, leave as 0 and unit as pixel
   2. Define unit (px or mm)
   3. Enter min and max plaque size and minimum circularity.

Note: for High resolution images we recommend a min plaque size of 300 to reduce false positives.

- 1. Enhance value can be changed (affects only Difference method).
  2. If Auto-thresholding box is checked step 5 will be omitted (affects only LowRes and Difference methods).
  3. If manual control box is unchecked step 6 will be omitted.

1. Use oval selection tool to select the well area to analyze.
2. Use threshold window to select the optimal segmentation and press apply. Then press OK.
3. Use ROI manager to control the plaques detected:
4. to discard false positives, select them and press delete.
5. to manually add more plaques, select them using the oval selection tools and press add.
6. when finished, press measure in the ROI manager window.
7. View and save results.

Instructions for running the macro ViralPlaque (method Weka)

1. Open image file to analyze.
2. To run the macro: Plugins 🡪 Macros 🡪 ViralPlaque
3. Select method Weka in popup. Additionally, enter settings values (if none is changed default values are used):
   1. Enter number of pixels per unit. If scale is not known, leave as 0 and unit as pixel
   2. Define unit (px or mm)
   3. Enter min and max plaque size and minimum circularity.

Note: for High resolution images we recommend a min plaque size of 300 to reduce false positives.

- 1. If manual control box is unchecked step 6 will be omitted.

1. Use oval selection tool to select the well area to analyze.
2. Indicate classifier file.
   1. Navigate to and select the classifier file, and click ‘Open’. A classifier file obtained from our data is provided for the user at: <https://sourceforge.net/projects/viralplaque/>
3. Alternatively, new classifiers can be trained and a new file can generated (please see further instructions in next page)
4. Use ROI manager to control the plaques detected:
5. to discard false positives, select them and press delete.
6. to manually add more plaques, select them using the oval selection tools and press add.
7. when finished, press measure in the ROI manager window.
8. View and save results.

Instructions for training new classifiers from user data (Trainable Weka Segmentation).

Follow these steps to obtain a new classifier file from user data image file.

1. Open image file to use as training data.
2. To run WEKA: Plugins 🡪 Segmentation 🡪 Trainable Weka Segmentation.
3. Use line tool to select pixels that correspond to stained cells and press add to class 1. This step can be repeated.
4. Use line tool to select pixels that correspond to viral plaques and press add to class 2. This step can be repeated.
5. In the settings menu: select training features. The less features the faster the training will be but that may impact in the precision of the future classification process. We recommend not to use more than 3 features.
6. Press train classifier and wait the process to finish (can take several minutes).
7. Use the toggle overlay to see if the classification is good enough. If not, select more pixels that correspond to the different classes and repeat step 6.
8. Finally, press save classifier to obtain the classifier file to use with the WEKA method.

For an example of usage see Supplementary video 4. For specific details on each step and the training features please see Trainable Weka Segmentation help at: https://imagej.net/Trainable_Weka_Segmentation

Instructions for running the macro ViralPlaque in 6-well mode.

1. Open image file to analyze.
2. To run the macro: Plugins 🡪 Macros 🡪 ViralPlaque
3. Select 6-well mode in prompt window. Additionally, enter settings values (if none is changed default values are used):
   1. Enter number of pixels per unit. If scale is not known, leave as 0 and unit as pixel
   2. Define unit (px or mm)
   3. Enter min and max plaque size and minimum circularity.
   4. If manual control box is unchecked step 6 will be omitted.
4. Use rectangular selection tool to select the well area to analyze. Be careful to select all the 6 wells. Image must be vertical. Please see Figure A for more details.
5. Indicate classifier file.
6. Navigate to and select the classifier file, and click ‘Open’. A file is provided. Alternatively, new classifiers can be trained and a new file can generated (please see Trainable Weka Segmentation help for specific use)
7. Use ROI manager to control the plaques detected:
8. to discard false positives, select them and press delete.
9. to manually add more plaques, select them using the oval selection tools and press add.
10. when finished, press measure in the ROI manager window.
11. View and save results.


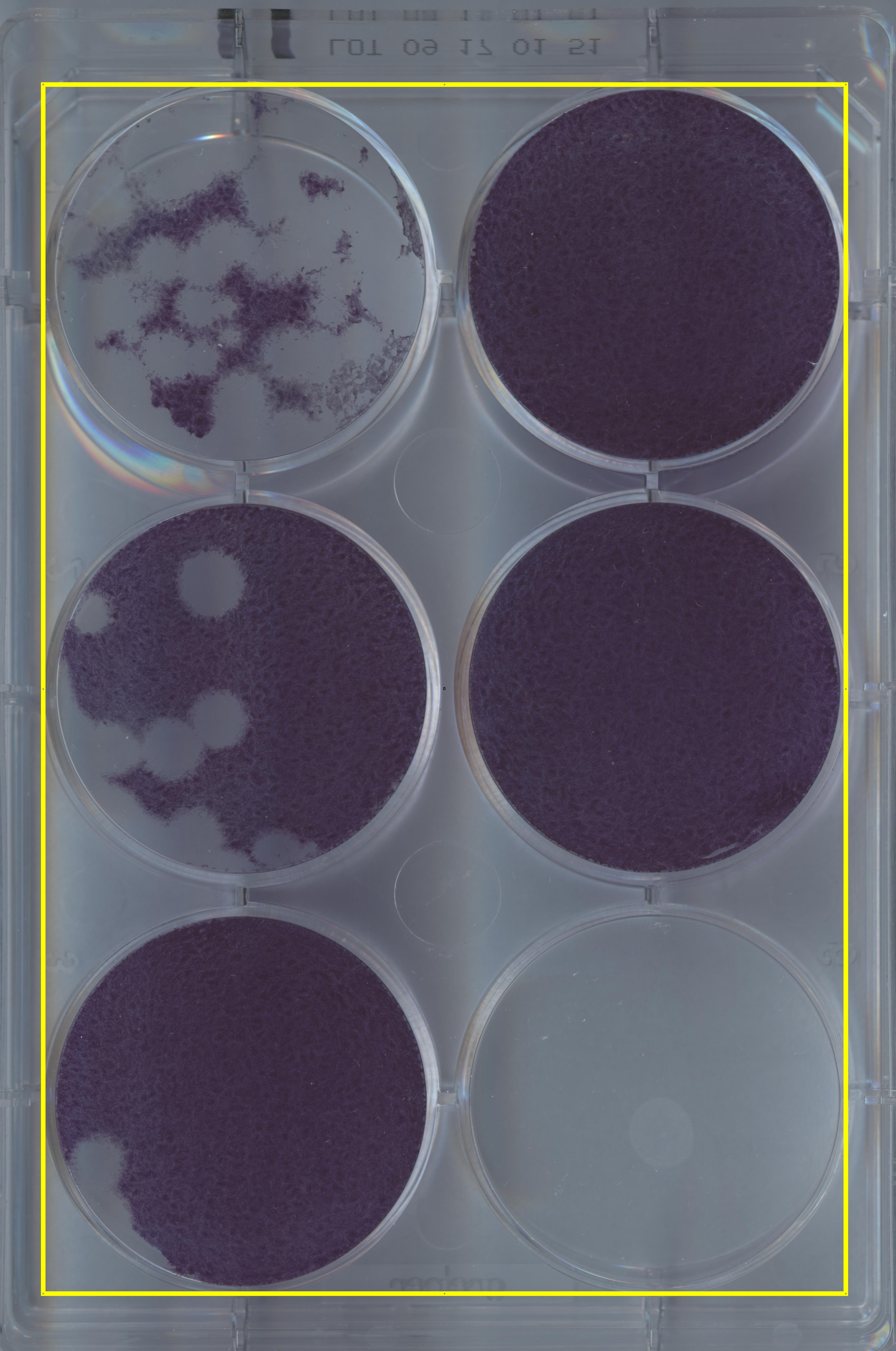


**Figure A Example of an image analyzed with 6-well mode.** Use rectangular selection tool (preselected in macro) to select all 6 wells. Plaque must be vertical (this can be done in ImageJ using Image 🡪 Transform 🡪 Rotate)

Instructions for running the macro ViralPlaque-batch mode.

1. Download and install the ViralPlaque-batch macro.
2. To run the macro: Plugins 🡪 Macros 🡪 ViralPlaque-batch
3. Navigate to and select the input directory (where the image files to analyzed are stored), and click ‘Open’
4. Navigate to and select the output directory (where the results files are to be saved), and click ‘Open’
5. ImageJ ViralPlaque macro will be run sequentially on every image on input directory.
6. Follow corresponding instructions for each image.
7. A results file name after the analyzed image will be automatically saved on the output directory.
8. Back to step 5a until all images in the input directory are processed.
